# Enhancing efficiency of a laboratory-scale hybrid cooling tower using Fe_3_O_4_-water nanofluid and spiral coils

**DOI:** 10.1016/j.heliyon.2024.e41370

**Published:** 2024-12-18

**Authors:** Danial Fallah Heravi, Hamid Reza Goshayeshi, Reza Saleh

**Affiliations:** Department of Mechanical Engineering, Mashhad Branch, Islamic Azad University, Mashhad, Iran

**Keywords:** Hybrid cooling tower, Spiral coil, Fe_3_O_4_ -water nanofluid, Secondary flow

## Abstract

This study presents an in-depth investigation into improving the efficiency of a laboratory scale hybrid cooling tower by utilizing Fe₃O₄-water nanofluid at varying mass fractions, ranging from 0.015 % to 0.15 %, along with different coaxial spiral coil configurations. The experimental setup includes three spiral coils with diameters of 15 cm, 25 cm, and 35 cm, and a pipe diameter of 14 mm. By analyzing the relationship between cooling tower efficiency and the Merkel number, this research establishes a quantitative correlation between these factors. The novelty of this study lies in its unique combination of Fe₃O₄-water nanofluid and the spiral coil geometries, a configuration that has not been explored in prior studies for enhancing heat transfer in hybrid cooling towers.

Experimental results indicate a significant 50 % improvement in cooling tower efficiency when Fe₃O₄-water nanofluid is used compared to pure water, largely due to enhanced thermal conductivity. Furthermore, the secondary flow generated by the spiral coils contributed an additional 8 % improvement in heat transfer. This work not only introduces a novel cooling tower design but also demonstrates the potential of nanofluids to significantly boost cooling efficiency in various industrial applications.

By optimizing heat transfer performance through advanced fluid and geometric configurations, this study provides a comprehensive framework for future innovations in energy-efficient cooling technologies.

Looking ahead, the research offers promising avenues for further exploration, such as optimizing nanofluid compositions, testing different nanomaterials or hybrid fluids, and exploring alternative tower configurations. The scalability of the proposed system presents strong potential for real-world industrial applications, driving the development of sustainable, energy-efficient cooling solutions in various sectors.

**Table:** Nomenclature

*q*_*w*_	Total heat transfer, bulk water to interface, kW
*L*	Inlet water mass flow rate, kg h^−1^
*K*_*L*_	Unit conductance, heat transfer, bulk water to interface, kcal h^−1^. M^−2^ K^−1^
*G*	air flow rate, kg_dry__air_ h^−1^
*V*	Cooling volume, m^*3*^
*a*	Area of the interface, m^2^ m^−3^
*t”*	Intermediate Temperature K
*t*_*a*_	Dry-bulb Temperature K
*c*_*p*_	Specific Heat Capacity
*qs*	Sensible heat transfer, interface to the airstream, kW
h''	Enthalpy of moist air at interface temperature, Btu lb^−1^_dry__air_
h_a_	Enthalpy of moist air, Btu lb^−1^_dry__air_
*K*_*G*_	Overall unit conductance, sensible heat transfer between interface and the main airstream, kcal h^−1^.m^−2^K^−1^
c_pm_	humid specific heat of moist air Btu lb^−1^ F^−1^
m	Mass transfer rate, interface to airstream, kg h^−1^
K'	Unit conductance, mass transfer, interface to the main airstream, kg h^−1^. M^−2^
W″	Humidity ratio of the interface (film), kg kg^−1^
*W*_*a*_	Humidity ratio of air, kg kg^−1^
*q*_*L*_	Latent heat transfer, interface to the airstream, kcal h^−1^
*r*	Latent heat of evaporation (constant), kW kg^−1^
$\mu_{bf}$	Viscosity of base fluid, N.S m^−2^
$\rho_{bf}$	Density of base fluid, kg m^−3^
EC	Electrical conductivity, ℳs cm^−1^
in	inlet
out	Outlet
np	Nanoparticle
Wt. %	Weight concentration
PVC	Polyvinyl chloride
HCT	Hybrid Cooling Tower
Hp	Horsepower
V _1~17_	Valve 1 to 17
$k_{nf}$	Conduction heat transfer of nanofluid, W m^−2^.K^−1^
$k_{bf\;}$	Conduction heat transfer of base fluid, W m^−2^.K^−1^
$\mu_{nf}$	The viscosity of nanofluid, N.S m^−2^
$\rho_{nf}$	The density of nanofluid, kg m^−3^
$C_{P_{nf}}$	Specific Heat Capacity of nano fluid
Me	Merkel number
T_w_	Temperature of water, K
T_wb_	Wet bulb Temperature, K
T_surface_	Surface Temperature of coils, K

## 1 Introduction

A cooling tower is a device designed to reduce the temperature of a circulating fluid, typically water, by dissipating heat into the atmosphere. The primary mechanism of cooling in these towers is the evaporation of water, which brings the working fluid close to the wet-bulb temperature. In closed-circuit or dry cooling towers, air alone is used for cooling, often utilizing cooling coils to bring the medium near the dry-bulb temperature. Hybrid cooling towers combine wet and dry cooling methods, employing evaporative effects on a secondary fluid outside the coils and sensible heat transfer from a primary coolant inside the coils.

Researchers have extensively investigated various aspects of cooling tower design, including types of packing and flow streams in wet cooling towers and the use of nanofluids to enhance heat transfer rates. Merkel's theory, developed by Franz Ernst Heinrich Merkel, is a critical framework for understanding heat and mass transfer in cooling towers. This theory provides a mathematical model for calculating heat transfer efficiency in wet cooling towers, considering factors such as temperature differences and water vapor pressure variations. It assumes that the cooling process adheres to heat and mass transfer principles, aiding engineers in estimating cooling capacity and performance to design and optimize cooling towers for industrial applications [[1], [2], [3], [4]].

Goshayeshi et al. [5] conducted an in-depth analysis of heat and mass transfer mechanisms in cooling towers, focusing on air-water interaction and droplet evaporation. Subsequent research has built on this foundation, exploring specific design and operational aspects of cooling towers. Studies have shown that the heat transfer coefficient decreases as the rib pitch-to-height ratio increases. For example, Xi et al. [6] found that straight wave packing in an experimental cooling tower increased cooling capacity by 14.4 % compared to conventional wave packing.

Sarker et al. [7] investigated hybrid cooling towers that blend wet and dry methods to enhance heat transfer efficiency. Their study revealed key design considerations and potential energy savings, particularly noting that hybrid cooling towers with finned copper tubes had a 22 % higher cooling capacity in dry mode and a 260 % higher capacity in wet mode compared to bare tubes. However, the finned tubes also experienced nearly double the pressure drop.

Another study investigated the application of nanofluids in cooling towers, utilizing nanoparticles like Fe₃O₄ to enhance heat transfer properties and overall performance. Imani-Mofrad [8] and colleagues examined various nanofluids at different concentrations in a bench-scale wet cooling tower, employing nanoparticles such as zinc oxide, silica, alumina, and graphene. Their findings indicated that graphene/water nanofluid was the most effective, improving cooling tower performance by 8.3 % and 36 %, respectively, compared to pure water. Conversely, alumina nanoparticles diminished performance by 14.7 %. Additionally, the use of zinc oxide/water nanofluid in different packings enhanced thermal characteristics by 21.5 % and 22.5 % at 0.02 % and 0.05 % mass fraction concentrations, respectively [8].

Sadafi [9] and collaborators conducted both experimental and simulation studies on an industrial cooling tower using Ansys Fluent to compare the effects of saline water versus pure water in a dry cooling tower. Their results demonstrated that saline water improved cooling performance and reduced both infrastructure and water costs compared to pure water.

Askari and colleagues [10] examined the conservation of energy and water in wet cooling towers by utilizing multi-walled carbon nanotubes (MWNTs) and nanoporous graphene nanofluids. They discovered that MWNTs exhibited higher thermal conductivity than nanoporous graphene. However, the cooling range increased by 40 % with MWNTs and by 67 % with nanoporous graphene compared to pure water [10].

Additionally, studies have investigated integrating renewable energy sources with cooling towers. Xie et al. [11] examined solar energy systems combined with cooling towers, emphasizing sustainable and energy-efficient cooling solutions. They found that aluminum oxide nanofluid increased the heat transfer rate by 22 % and reduced water consumption by 15 % in a laboratory-scale closed wet cooling tower compared to pure water [11].

Sharghway et al. [12] developed a numerical model for a wet cooling tower using seawater as the working fluid, identifying a correction factor based on seawater salinity and temperature.

Sun et al. [13] utilized a natural draft dry cooling tower as a heat sink for a concentrated solar power plant, experimenting with various spray cooling designs. Their findings indicated that pre-cooling inlet air enhanced cooling tower effectiveness and reduced electrical power consumption.

Mofrad et al. [8] developed a method to standardize ambient air conditions in wet cooling towers by utilizing various nanofluids. They found that a graphene/water nanofluid at a concentration of 0.02 wt% provided the highest thermal efficiency, boosting volumetric heat transfer by 36.2 % compared to pure water.

Abolfazli et al. [14] explored the effects of cooling towers on the performance of oscillating heat pipes, determining that a 160-kW cooling tower paired with an eight-pass oscillating heat pipe achieved optimal results.

Ge et al. [15] conducted theoretical studies on natural draft dry cooling towers, examining factors such as tower shape, crosswind impact, and radiator configurations. They concluded that optimizing tower height could reduce construction costs, with tower shape being the most significant factor.

Wei et al. [16] created a mathematical model for experimental cooling towers in a 600 MW power plant, studying the impact of variables such as air properties and water mass flow rate on performance. Their results indicated that the water mass flow rate had the least effect on the evaporation rate, whereas a decrease in dry bulb temperature led to a reduction in the evaporation rate.

In another study, Wei et al. [17] theoretically analyzed a natural draft hybrid cooling tower used in the power generation industry. Using a physico-mathematical model, they predicted the cooling system's performance, revealing that natural draft cooling systems equipped with wet sections can mitigate the disadvantages of wet cooling towers, especially in arid regions.

Advancements in computational modeling and simulation techniques have furthered the understanding and optimization of cooling tower performance. Numerous studies have utilized numerical simulations to analyze fluid flow patterns, heat transfer characteristics, and the impact of different design parameters on the efficiency of cooling towers [14,18,19].

Purbia et al. [18] conducted an economic evaluation and mathematical modeling of graphene/water nanofluid in heat exchangers. Using MATLAB, they developed an energy-efficient cooling system model. Their simulations, which included nanoparticle concentrations ranging from 0.025 % to 0.1 %, suggested that graphene nanofluid enhances the thermal performance of heat exchangers and significantly reduces operating costs compared to conventional nanoparticles such as TiO₂ or Al₂O₃.

Monjurul et al. [20] developed a MATLAB code to design an NDDCT for a 25 MW solar plant using supercritical CO2 Brayton cycle, specifying dimensions and heat exchanger bundles. Performance is analyzed for varying temperatures and pressures, showing reduced cooling efficiency during high ambient temperatures. They have also investigated dry cooling technology, particularly dry natural draft cooling towers (NDDCT), as an alternative to wet cooling in arid regions, applied to supercritical CO2 power cycles. Their study includes designing an NDDCT for a 25 MW solar plant to optimize efficiency under varying conditions. They find the recompression cycle offers superior performance and cost-effectiveness, especially in extreme ambient temperatures, making it ideal for future commercialization.

Joshi et al. [21]developed a chilled water system analysis tool that quantifies energy consumption in various systems, including cooling towers, using typical measurements and experimental data.

Yu et al. [22] compared a novel combined heat exchanger strategy with conventional wet cooling towers to reduce evaporation rates and pollution. They found that using combined heat exchangers can save up to 21.5 % of annual water consumption by lowering water temperature.

Recent research has also focused on the environmental impact and sustainability of cooling towers. Rahmati et al. [23] and Ma et al. [24] investigated water consumption and environmental implications, proposing strategies to reduce water usage and improve sustainability.

Rahmati [23] examined the impact of zinc oxide nanoparticles and varying packing densities on the performance of wet cooling towers. The study revealed that increasing the packing density boosts both the water temperature difference and overall cooling tower efficiency. Additionally, higher-density packing enhances the effectiveness of nanofluids.

Guo et al. [25] developed a mechanistic model based on Merkel's theory and Least Squares Support Vector Machine to account for variations in outlet water temperature due to changes in operating conditions in hybrid cooling towers. Their simulations, utilizing the Gaussian Mixture Model (GMM), showed that the parallel hybrid model offers better prediction accuracy compared to the mechanistic model. They also created a fast and accurate analytical method for heat and mass transfer modeling in cooling tower energy systems, comparing experimental and analytical results to determine temperature distribution across different sections of the tower.

Awais et al. [26,27] provide a thorough review of nanofluids, emphasizing their superior heat transfer properties compared to conventional fluids. They discuss how factors like nanoparticle size, shape, and concentration, along with mechanisms such as Brownian motion and thermophoresis, impact thermal conductivity and transport properties. The study also highlights the importance of proper measurement techniques and modeling to optimize nanofluid use in industrial applications. Additionally, nanofluids exploration in high-performance heat transfer systems like HVAC and electronics cooling, noting challenges such as surface fouling and pressure drop is investigated.

Ma et al. [24] investigated the energy and environmental impacts of various packing materials, with a focus on bamboo grids. They found that while PVC-type packing delivers higher thermal performance, substituting PVC with bamboo grids significantly reduces total energy consumption in power generation plants. Energy consumption with PVC cooling towers was found to be six times higher than with bamboo grids.

Javadpour et al. [28,29] studied the cooling performance of an experimental cross-flow cooling tower using a water/MWCNTs nanofluid. Their findings showed that the cooling tower's effectiveness with nanofluid was 10.2 % higher than with pure water. They also discovered that large grid splash-type fillers were the most effective, increasing cooling tower effectiveness by approximately 28 %.

Karimi et al. [30] aimed to enhance the thermal performance of a cross-flow cooling tower (CFWCT) by using nanofluids with improved thermal properties and reduced water loss. They tested MWCNTs/H₂O, MWCNTs-COOH/H₂O, and MWCNTs-OH/H₂O nanofluids as replacements for water. The research indicated that functionalized nanofluids have lower evaporation rates than water, while non-functionalized nanofluids have higher evaporation rates.

In conclusion, scientific research on cooling tower technology provides crucial insights into design, optimization, and performance enhancement, contributing to the development of more efficient, sustainable, and environmentally friendly cooling tower systems [29].

Hassab et al. [31] introduced a new correlation linking cooling tower effectiveness to the number of transfer units (NTU), streamlining the simulation of thermal performance and design for counterflow cooling towers (CCCT). This approach simplifies the process by avoiding the iterative methods typically used. The study's outcomes, validated against experimental data, showed excellent alignment—differences were within ±5 % of the authors' experimental results and ±2 % of Jiang et al.'s findings [32,33].

Navaro et al. [34] worked on enhancing the thermal efficiency of a prototype forced mechanical-draft, wet, inverted cooling tower. They investigated the impact of fill length and nozzle configuration on performance, noting very low particle emissions. Their results indicated that an upper manifold with parallel flow outperformed the intermediate and lower manifolds thermally. Additionally, a fill length of 1.6 m proved superior to other tested lengths. These findings suggest that uniform flow combined with a larger heat exchange surface area offers the best setup for improved cooling tower efficiency. They also evaluated a cooling tower designed to minimize atmospheric pollutant dispersion during operation, discovering that the Poppe method yielded higher Merkel Numbers compared to the Merkel method. They recommended using a combination of counterflow and parallel flow arrangements with the Poppe method for thermal performance evaluation [35].

Zhao et al. [36] investigated the effects of corrugated film-packing structures in cross-flow cooling towers. They found that optimal packing structures enhanced cooling capacity by 3.8 %–12.2 %. Moreover, they established empirical formulas for the thermal and resistance characteristics of various packing types. Long et al. [37] examined the resistance and splash performance of water collection devices in mechanical draft cooling towers, revealing that different water collector designs significantly influence pressure drop.

Sobecki et al. [38] conducted a study using image analysis and computational modeling techniques to estimate the power output of a mechanical draft cooling tower. They captured plume images with multiple cameras and used advanced algorithms to quantify the volumes of condensed water vapor plumes. They developed a one-dimensional code incorporating weather data, tower operating conditions, and plume volume measurements to compute power output. The model was validated against observed data and showed good agreement, with an average error of 6 %–12 %. The study highlights the potential of using visible imagery to determine power plant fuel consumption rates.

Ahmad et al. [39,40] analyzed the impact of nanofluids and hybrid nanofluids on heat transfer and pressure drop in double-dimpled corrugated pipes, finding a 20–25 % heat transfer improvement over smooth pipes. Using computational fluid dynamics, they observed the best performance with a 3 % Al₂O₃-CuO/water hybrid nanofluid, achieving a 20.62 % thermal efficiency increase. They also explore various corrugated mini-channels, demonstrating a 25–30 % enhancement in heat transfer coefficients compared to smooth channels. The highest performance, a 22.19 % increase, was found in a rectangular channel with a specific corrugation design, confirming that corrugated designs significantly boost heat transfer efficiency.

Ahmad et al. [41] show that corrugated mini-channels (rectangular, sine-shaped, and V-shaped) enhance heat transfer by 25–30 % compared to smooth channels, with the best performance from a 3 % Al₂O₃-CuO/water mixture.

Yu et al. [42] investigated the airflow distribution in a hybrid cooling tower with a parallel path configuration. Their study revealed that modifying the airflow path could enhance overall cooling efficiency. Li et al. [43] further improved upon this by adding baffles to the distribution pipe surface, which significantly improved airfield uniformity within the cooling tower. The addition of a1a2-type baffles notably increased air velocity in the packing zone, thereby reducing both air and circulating water temperatures in the tower's central area and enhancing cooling effectiveness compared to conventional designs.

Zhou et al. [44] concentrated on the heat transfer efficiency of elliptical-tube heat exchangers in closed-circuit cooling towers. Through a combination of experiments and simulations, they compared heat transfer under various operating conditions and analyzed the effects of parameters such as wind speed and spray water flow rate on flow field characteristics and heat transfer performance. Their findings showed that optimized deflected elliptical tubes achieved a 16.27 % increase in the heat transfer coefficient compared to standard configurations.

Mustakim et al. [45] focused on analyzing the flow and heat transfer in helically corrugated pipes to assess the performance of single and hybrid nanofluids at different Reynolds numbers. The study examines how changes in the helical pitch and the shape of the corrugation at the inlet affect heat exchanger efficiency by using ANSYS software. The results revealed that altering the corrugation shape at the inlet significantly improved performance, with the pipe featuring the smallest pitch showing the best results. The study found that the heat transfer coefficient in pipes with the tested nanofluids was 20–30 % higher than in smooth pipes. The aim of this research is to enhance the efficiency of a laboratory-scale hybrid cooling tower by utilizing Fe₃O₄-water nanofluid and different coaxial spiral coil configurations. The study seeks to investigate the combined effects of nanofluid-enhanced heat transfer and the secondary flow generated within spiral coils, aiming to significantly improve heat transfer performance, reduce energy consumption, and optimize cooling tower designs for industrial applications.

Despite advancements in cooling tower research, gaps remain in understanding how nanofluids and varying heat exchanger designs affect cooling efficiency, especially in hybrid systems. Previous studies focused on individual nanoparticle types or cooling methods without exploring the interaction between nanofluids and tower geometries. This paper addresses these gaps by introducing a novel hybrid cooling tower design incorporating Fe₃O₄-water nanofluid and coaxial spiral coils with different geometries. The study investigates the combined effects on heat transfer, the impact of secondary flow within spiral coils, and the potential to reduce energy consumption. The novelty lies in the simultaneous exploration of nanofluid-enhanced heat transfer and spiral coil geometries, which have not been studied previously. The research offers insights into optimizing hybrid cooling towers for industrial applications and improving energy efficiency in systems such as power generation, HVAC, and chemical processing. By using Fe₃O₄ nanoparticles in water-based nanofluid and spiral coils, this approach could significantly enhance thermal management in industrial cooling processes.

This study is a laboratory-scale investigation, and as such, a formal cost analysis is not included. However, based on the experimental results, several potential cost benefits are estimated. We anticipate **cost reduction, sustainability**, and **higher efficiency** as key advantages of this innovative cooling tower system.

## 2 Cooling tower theory

**Fig. 1:**
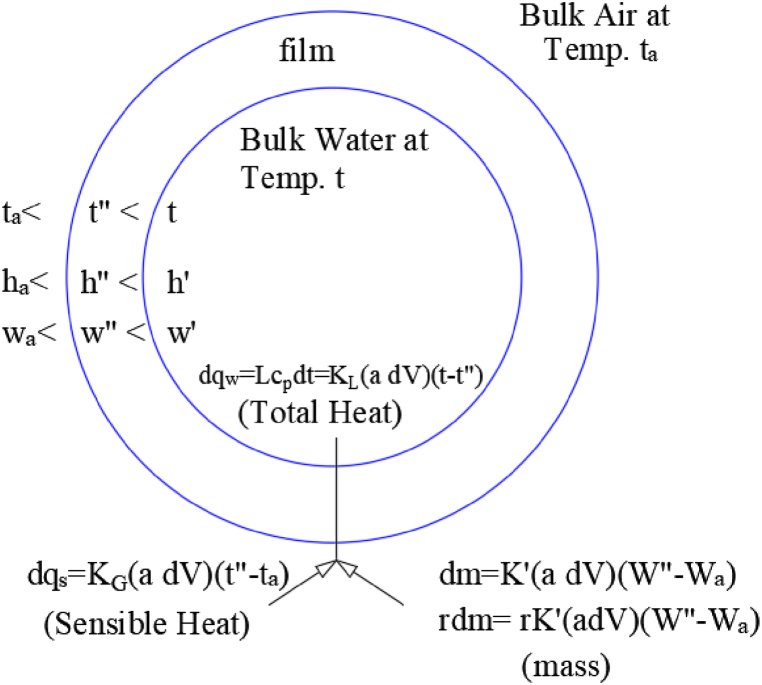
Heat and mass transfer relationship between water, interfacial film, and air [4].

Baker and Shryock introduced a detailed theory [4] explaining the processes within a cooling tower. Consider a cooling tower with a one-square-foot base, a cooling volume V, and an extended water surface area per unit volume a. Let L denote the water mass flow rate, and G signify the air mass flow rate. Fig. 1 presents a schematic illustration of the mass and energy transfer processes.

In this setup, the bulk water at temperature t is surrounded by bulk air at a dry-bulb temperature t_a_, with an enthalpy h_a_ and a specific humidity ratio. The interface is conceptualized as a film of saturated air with an intermediate temperature t′′, enthalpy h′′ and humidity ratio W′′ [4]. The total energy transfer from water to the interface is represented mathematically in Equation (1). This forms the basis for analyzing heat transfer efficiency in the cooling tower [4].

(1) $${dq}_{w}=Lc_{p}\;dt=K_{L}\;a\left(t-t^{\prime \prime }\right)dV$$

The heat transfer from the interface to air, described by Equation (2), accounts for sensible heat transfer, which is crucial for understanding energy dynamics in the system [4].

(2) $$dq_{s}=K_{G}a\left(t^{\prime \prime }-t_{a}\right)dV$$

The water vapor diffusion from the film to air, a process critical in cooling tower operation, is detailed in Equation (3) [4].

(3) $$dm=K^{\prime }a\left(W^{\prime \prime }-W_{a}\right)dV$$

Equation (4) quantifies the heat transfer due to evaporation, a primary mechanism in hybrid cooling towers [4].

(4) $$dq_{L}=rdm=rK^{\prime }a\left(W^{\prime \prime }-W_{a}\right)dV$$

The process equilibrium is reached when t_a_ = t, and the air is saturated with moisture at that temperature. In adiabatic conditions, equilibrium is achieved at the adiabatic saturation temperature or the thermodynamic wet-bulb temperature of the air, which is the lowest temperature achievable in a cooling tower. When the tower operates without a heat load, the circulating water quickly reaches this temperature. When a heat load is applied, the process remains the same, but the air enthalpy increases as it moves through the tower, gradually raising the equilibrium temperature. The cooled water's approach to the entering wet-bulb temperature depends on the tower's efficiency. Merkel assumed the Lewis relationship to be one, integrating the mass and sensible heat transfer into a single coefficient driven by the enthalpy difference [4,35,46]. Equation (5) incorporates the Lewis relationship, simplifying the combined heat and mass transfer analysis for adiabatic conditions [4].

(5) $$\frac{K_{G}}{K^{\prime \;}c_{pm}}=1$$

The relationship between water heat loss and air heat gain is expressed in Equation (6), providing insights into system equilibrium [4].

(6) $$Lc_{p}dt=G\;dh=K^{\prime }a\left(h^{\prime \prime }-h_{a}\right)dV$$

This equation accounts for the transfer from the interface to the airstream, even though the interfacial conditions are not defined. By assuming negligible film resistance and using an overall coefficient K′ based on the enthalpy difference at the bulk water temperature t, the equation is transformed into equations (7), (8). For practical applications, Equation (7) transforms the heat transfer model using an overall coefficient, streamlining the analysis. The differential equations for bulk water temperature and enthalpy transfer are consolidated in Equation (8), enabling a simplified evaluation of cooling performance [4].

(7) $$\frac{K^{\prime }aV}{L}=\int_{t_{1}}^{t_{2}}\frac{c_{p}}{(h^{\prime }-h_{a)}}dt$$

(8) $$\frac{K^{\prime }aV}{G}=\int_{h_{1}}^{h_{2}}\frac{dh}{(h^{\prime }-h_{a)}}$$

In hybrid cooling towers, the Merkel number evaluates the overall cooling performance by accounting for both internal heat transfer (within the working fluid's closed circuit) and external evaporative cooling (outside the circuit). The type of working fluid, whether pure water or nanofluids, influences the heat transfer properties and, consequently, the Merkel number, which affects the cooling tower's efficiency. Equation (9) introduce Merkel number in evaporative cooling towers. Equation (10) provides a formulation for the Merkel number, a dimensionless parameter critical in assessing hybrid cooling tower efficiency [4,46].

(9) $$Me=\int_{T_{w}^{out}}^{T_{w}^{in}}\frac{dT_{w}}{T_{w}-T_{wb}}$$

(10) $${Me}_{HCT}=\int_{T_{w}^{in}}^{T_{w}^{out}}\frac{dT_{w}}{T_{wb}-T_{surface}}$$

As the integral does not have a simple analytical solution, approximation is used by means of the trapezoidal rule by dividing range of T_w_ in to small intervals.

## 3 Experimental setup

This study focused on designing and setting up a complex laboratory-scale hybrid cooling tower (HCT). The HCT comprises various components, including pumps, an axial fan, spiral and flat coils, an electrical heater, porous media, water distribution nozzles, a heat exchanger, measuring instruments such as digital thermometers, pressure gauges, a digital water flow meter, humidity meters, different types of valves, and piping with various flow configurations to achieve both series and parallel pipe arrangements.

**Fig. 2:**
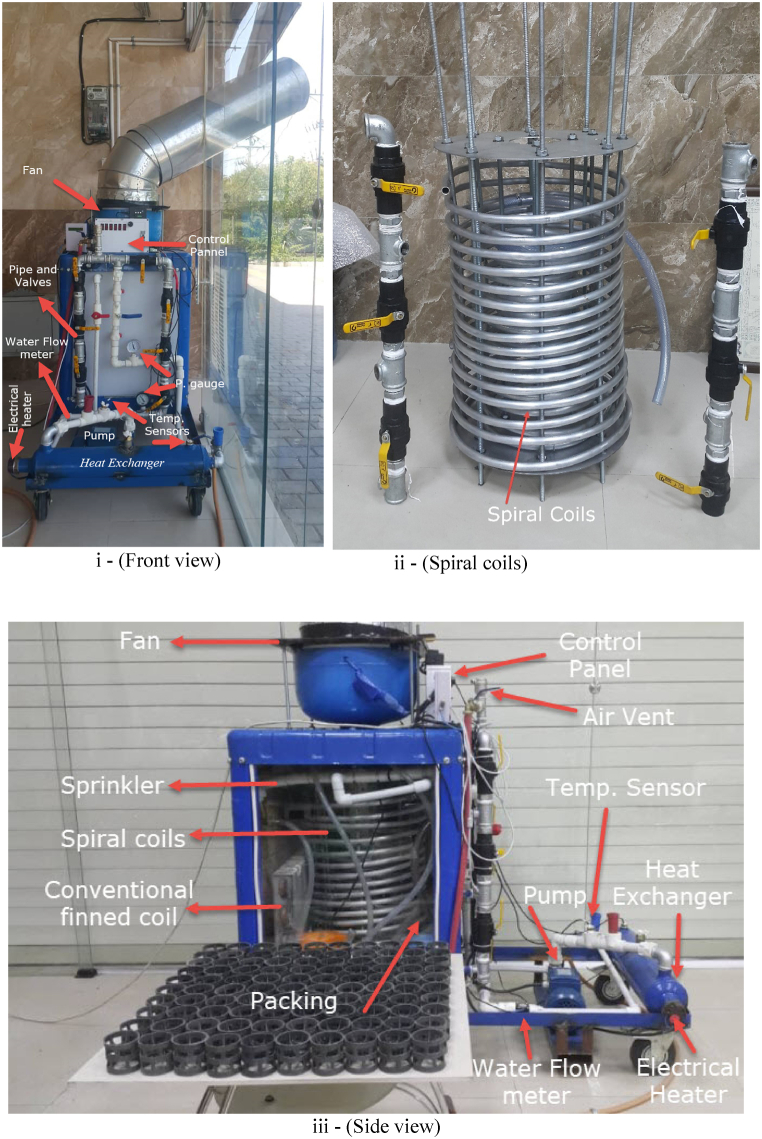
Experimental Setup, (i): front view, (ii): 3 spiral coils - (Da = 35 cm, Db = 25 cm, Dc = 15 cm), (iii): side view.

Fig. 2 illustrates the final experimental setup, where three coaxial, non-finned spiral coils are connected to a collector and a conventional finned coil with valves. In which, panel (i) showing the front view, panel (ii) highlighting the spiral coils, and panel (iii) presenting the side view. The setup features both a closed and an open cycle. The closed cycle starts with a 0.5 Hp centrifugal pump. The fluid then flows through a heat exchanger equipped with an electric heater, controlled by a digital thermostat, and monitored by two DS18B20 thermometers at the heat exchanger's inlet and outlet. Adjacent to the heat exchanger, a pressure gauge and a YF-S201 water flow meter sensor are installed in the pipeline. To ensure accurate flow rate measurements, the entrance pipe length to the flow meter is more than ten times its diameter. The cooling tower cycle flow rate can be controlled by two methods: using valves or a potentiometer in the pump's electrical circuit.

The next segment of the cycle includes a pipe collector and valves connected to aluminum coaxial spiral coils, allowing flow direction control. Two DS18B20 thermometers are installed at the collectors' inlet and outlet. A flat finned coil is also installed at the cooling tower exit with a pressure gauge to capture the fin effects on cooling tower performance and characteristics.

The open part of the hybrid cooling tower consists of two small submersible pumps. One pump circulates fluid from the cooling tower basin to the upper nozzles, wetting both spiral and flat coils. The other pump humidifies the airflow through porous media. Ambient air flows through porous media, cooling tower packing, and coils using an induced fan mounted on top of the cooling tower. The air inlet and outlet temperature and humidity are measured to determine the water evaporation rate.

From a dimensional perspective, the heat transfer control volume of the cooling tower is a box with a 60 cm × 60 cm cross-section and a height of 180 cm. The lengths of the inner, middle, and outer spiral coils are 8 m, 12 m, and 18 m, respectively, with an internal spiral aluminum coil diameter of 14 mm. The spiral diameters are 15 cm, 25 cm, and 35 cm, with a spiral pitch of 15 mm. The spiral coils are arranged in a triangular configuration to maximize the heat transfer surface area. The internal diameter of the flat-finned coil is 14 mm, with a fin arrangement of 12 fins per inch and a total length of 3.2 m, including 8 standard elbows.

**Fig. 3:**
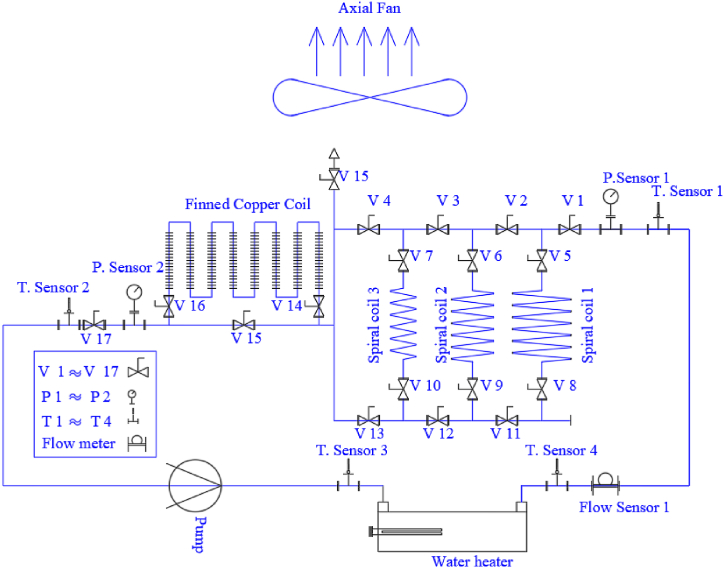
General flow diagram.

**Fig. 4:**
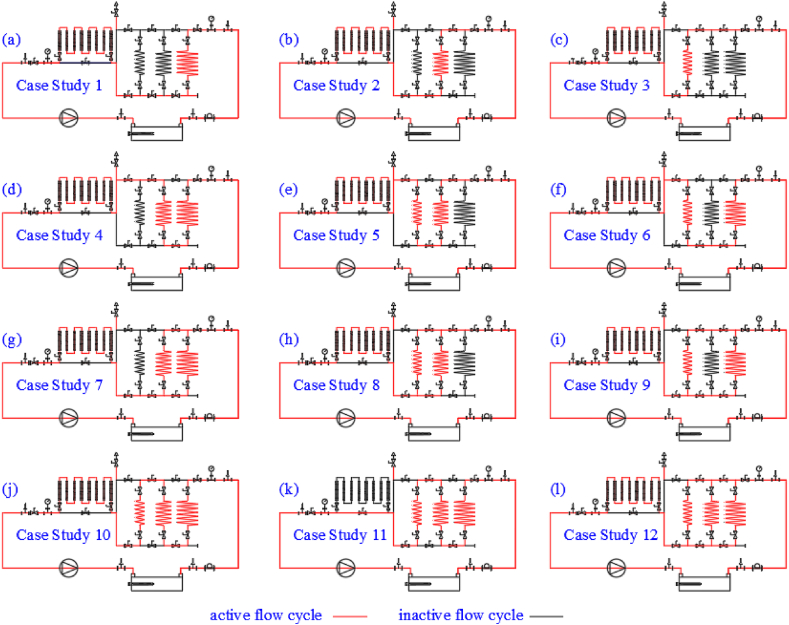
Flow diagram and directions in 12 different case studies - (a): Spiral Coil 1 is paired with a finned tube. (b): Spiral Coil 2 and a finned tube. (c): Spiral Coil 3 and a finned tube. (d): Spiral Coil 1 & 2 in series configuration and a finned tube. (e): Spiral Coil 2 & 3 in series configuration and a finned tube. (f): Spiral Coil 1 & 3 in series configuration and a finned tube. (g): Spiral Coil 1 & 2 in parallel configuration and a finned tube. (h): Spiral Coil 2 & 3 in parallel configuration and a finned. (i): Spiral Coil 1 & 3 in parallel configuration and a finned tube tube. (j): Spiral Coil 1, 2 & 3 in series configuration and a finned tube. (k): Spiral Coil 1, 2 & 3 in series configuration without a finned tube. (l): Spiral Coil 1, 2 & 3 in parallel configuration and a finned tube.

Fig. 3 depicts the general flow diagram and the equipment of the cycle, while Fig. 4 shows twelve different case studies, each serving as an independent experiment. Panel (a) represents the flow path configuration for Case Study 1, where Spiral Coil 1 is paired with a finned tube. Panel (b) illustrates Case Study 2, which uses Spiral Coil 2 and a finned tube, and so on through panel (l), which depicts the flow path for Case Study 12 involving Spiral Coils 1, 2, and 3 in a parallel configuration with a finned coil. These configurations highlight the versatility of the experimental setup in exploring series and parallel flows, as well as the effects of finned versus non-finned coils. The physical details of the spiral and conventional coils are provided in Table 1.

**Table 1:** Cooling tower flow diagram details at different flow paths.

Case study	Flow path	Equivalent length(m)
1	Spiral coil 1 & finned tube	18
2	Spiral coil 2 & finned tube	12
3	Spiral coil 3 & finned tube	8
4	Spiral coil 1 & 2 in series flow	30
5	Spiral coil 2 & 3 in series flow	20
6	Spiral coil 1 & 3 in series flow	26
7	Spiral coil 1 & 2 in parallel flow	15
8	Spiral coil 2 & 3 in parallel flow	10
9	Spiral coil 1 & 3 in parallel flow	13
10	Spiral coil 1 & 2 & 3 in series flow with finned coil	41
11	Spiral coil 1 & 2 & 3 in series flow without finned coil	38
12	Spiral coil 1 & 2 & 3 in parallel flow with finned coil	16

### 3.1 Boundary conditions

When conducting experimental analysis of a hybrid cooling tower, defining boundary conditions is essential to ensure accurate and reproducible results. Here are key boundary conditions to consider:

**Table:** 

Parameter	Condition	Type
Fluid Inlet Temperature to the electric heater	24 °C (initial, matches ambient temperature)	Ambient Conditions
Fluid Inlet Temperature to the cooling tower	34–40 °C	Initial Condition
Fluid Flow Rate	6 L/min (adjustable with valves, potentiometer)	Boundary Condition
Air Inlet Temperature	30 °C (constant under laboratory condition)	Initial Condition
Air Inlet Humidity	30 % (constant under laboratory condition)	Initial Condition
Nanofluid Mass Fraction	1.5 %–15 % (Fe₃O₄-water nanofluid)	Boundary Condition
Heater Set Temperature	40 °C (controlled with thermostat)	Boundary Condition
Heater Cutoff	±2 °C from set temperature	Boundary Condition
Steady State Achievement Time	30 min for nanofluid, 1 h for pure water	Initial Condition
Evaporation Rate	Monitored through air humidity change	results
Flow Configuration	Series, parallel, and mixed configurations	Boundary Condition
Cooling Tower Geometry	Spiral and flat coils, arranged in triangular setup	Initial Condition

### 3.2 Nanofluid preparation

**Fig. 5:**
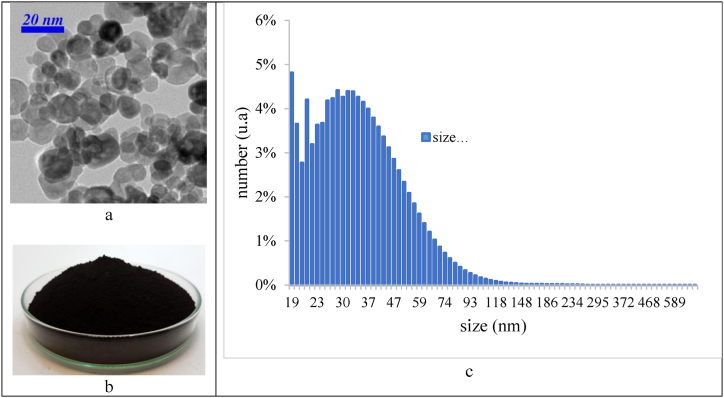
(a) SEM image of Fe_3_O_4_ nanoparticles. (b): sample of Fe_3_O_4_ nanoparticles. (c): Size dispersion of Fe₃O₄ nanoparticles.

In this study, nanofluids were prepared using the two-step method commonly cited in the literature [41]. The base fluid was deionized water, and the nanoparticles were Fe₃O₄. Fig. 5 shows the size dispersion and SEM image of the nanoparticles. panel (a) shows the SEM image, panel (b) depicts the sample of Fe₃O₄-nanoparticles and panel (c) provides the size dispersion data. Initially, the mixture was stirred with a mechanical mixer for about 30 min, followed by ultrasonication for 60 min [47]. The primary purpose of using nanofluids in heat transfer projects is to increase the overall heat transfer coefficient. However, the prepared nanofluid must first be stabilized. According to other studies, TMAH (Tetramethylammonium hydroxide) is used as a surfactant to achieve stability [48,49].

The following equations define the properties of the nanofluid [48]. The effective density of the nanofluid is calculated using Equation (11), incorporating the nanoparticle volume fraction. Equation (12) defines the effective specific heat capacity, critical for analyzing thermal performance. Equation (13) is utilized to determine the nanofluid's thermal conductivity, a key property for heat transfer enhancement. The effective viscosity, which impacts flow characteristics, is described in Equation (14) [48].

(11) $$k_{nf}=k_{bf\;}{\left(1+0.5\varphi \right)}^{0.1051}$$

(12) $$\mu_{nf}=\mu_{bf}{\left(1+\frac{\varphi }{12.5}\right)}^{6.356}$$

(13) $$\rho_{nf}=\left(1-\varphi \right)\rho_{w}+\varphi \rho_{{Fe}_{3}O_{4}}$$

(14) $$C_{P_{nf}}=\left(1-\varphi \right)C_{PW}+{\varphi C}_{P{Fe}_{3}O_{4}}$$

**Fig. 6:**
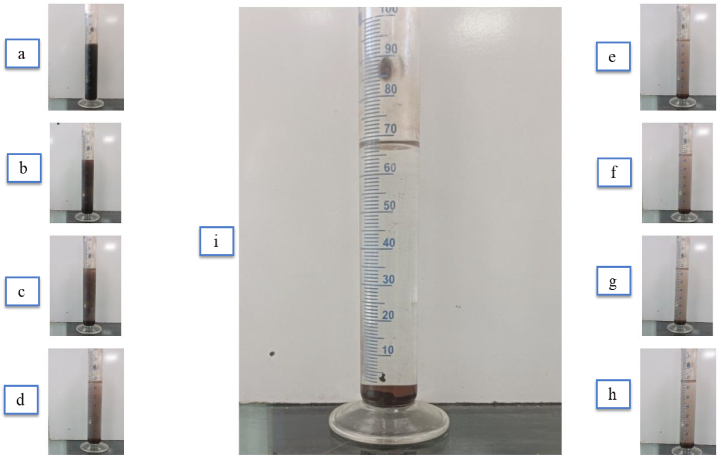
Settlement process of the nanoparticles. (a): sample from the cooling tower after the experiment; b: sample after ultrasonication; c to h: samples from the cooling tower every 12 days; i: nanofluid without surfactant showing complete settlement).

The current experiments were conducted over six months, and without surfactants, it was not feasible to maintain nanofluid stability throughout this period. Fig. 6 illustrates the instability of the nanofluid without a surfactant. The stability of the Fe₃O₄-water nanofluid over time is demonstrated in Fig. 6, with panels (a–h) showing the progression of settlement every 12 days and panel (i) highlighting the complete settlement of the nanofluid without a surfactant. Therefore, TMAH was used to stabilize the working fluid in the cycle. Summary of Effects of TMAH on the stability of Fe₃O₄-Water Nanofluid is listed in Table 2:

**Table 2:** Effects of TMAH on the stability of Fe₃O₄-Water Nanofluid.

Property	Effect of TMAH
Stability	Significantly improved; prevents agglomeration and sedimentation
Zeta Potential	Increased, leading to better electrostatic repulsion
Viscosity	Slightly increased due to particle coating
Thermal Conductivity	Enhanced due to better dispersion of nanoparticles
Magnetic Properties	Retained with more uniform particle response to magnetic fields
Specific Heat Capacity	Minimal change, as TMAH is typically used in low concentrations

**Fig. 7:**
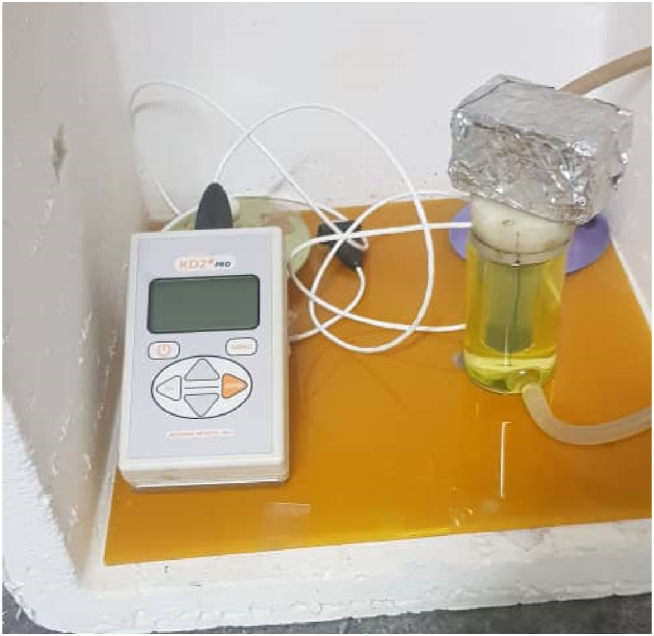
KD2 conduction heat transfer coefficient analyzer.

To ensure the stability of the working fluid during the experiments, the conduction heat transfer coefficient and electrical conductivity of the nanofluid were analyzed using a "KD2 Pro" thermal conductivity analyzer and an electronic EC meter, respectively. Fig. 7 shows the schematic of the KD2 analyzer. Results indicated that the nanofluid without a stabilizer settled after 2 h. Consequently, the thermal and electrical conductivity coefficients were investigated and analyzed at three different times: immediately after ultrasonication, an hour and a half after the test began, and after complete settlement.

**Fig. 8:**
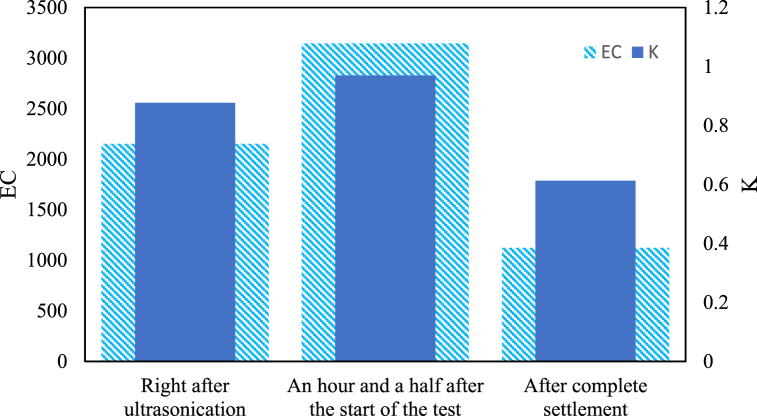
Comparison of thermal conductivity (Wm^−1^. K^−1^) and electrical conductivity (ℳs cm^−1^) in nanofluid.

The comparison of electrical conductivity and conduction heat transfer coefficient, depicted in Fig. 8, illustrates that the conduction heat transfer coefficient during the test is 58 % higher than after complete settlement and 10 % higher than immediately after ultrasonication. This indicates that the centrifugal pump installed in the system acts as a high-shear mixer, and the new design of the cooling tower with spiral coils prevents nanoparticle settlement. Thus, the secondary flow in the spiral coils and the centrifugal pump are the main physical mechanisms stabilizing the nanofluid during the experiment.

Fe₃O₄ nanoparticles utilized in this research were procured from Armina Nano Chemical Institute. The thermal properties of Fe₃O₄ nanoparticles are shown in Table 3. And physical properties of Fe_3_O_4_ nanoparticles that are used in this research are listed in Table 4.

**Table 3:** Thermal properties of magnetic nanoparticles.

Nanofluid	Density (gr/cm^3^)	Specific heat (J kg^−1^K^−1^)	Thermal conductivity (W m^−1^K^−1^)	Molecular weight (g/mol)	Magnetization Emu/g
Magnetic nanoparticles (Fe_3_O_4_)	5180	670	80	233.54	51.1

**Table 4:** – Physical properties of Fe_3_O_4_ nanoparticles.

Molecular formula	Fe3O4
Molecular weight (g/mol)	233.54
Form	Powder
Color	Black
Morphology	Spherical and cornered shape Crystal structure
Cubic Size range (nm)	30–40 nm
Magnetization	51.1 emu/g at 14 kOe
Curie temperature (°C)	585
Density (g/cm3)	8.9
Impurity by XRF (%)	<2

## 4 Uncertainty analysis

Uncertainty analysis in experimental studies involves evaluating and quantifying the potential errors and variations associated with measurements and results. It is crucial for understanding the reliability and precision of experimental data [50]. In this research, the combined uncertainty of instruments and repeatability of the experiments are investigated in equation (15) [50]:

(15) $$U_{Ri}=\frac{x_{i}}{R}\frac{\partial R}{\partial x_{i}}U_{xi}$$

where *x* is a Measurable parameter, *R* is a quantity calculated from measurable parameters, *U*_*xi*_ is measurement error for the experimental measured parameter and is equal to: (measurement accuracy)/(minimum measured value), and *U*_*Ri*_ is the Maximum possible error in calculating a quantity. The uncertainties of the parameters used in this research are given in Table 5.

**Table 5:** The uncertainties of instruments.

Parameter	*T*_*in*_	*T*_*out*_	*M˙*
Uncertainty	0.144	0.144	0.029

The combined maximum uncertainty of the experiment is expressed in equation (16) [50].

(16) $$\mathrm{Max}.\;U_{R}=\pm {\left[{\left(\frac{x_{1}}{R}\frac{\partial R}{\partial x_{1}}U_{x1}\right)}^{2}+{\left(\frac{x_{2}}{R}\frac{\partial R}{\partial x_{2}}U_{x2}\right)}^{2}+...+{\left(\frac{x_{n}}{R}\frac{\partial R}{\partial x_{n}}U_{xn}\right)}^{2}\right]}^{1/2}$$

In Eq. (9), UR is the maximum uncertainty intervals of final parameters in all values of xi (i = 1, 2, … n). Therefore, the maximum uncertainty for the experiment is ±3.6 %.

## 5 Results

The experiment consists of 12 different flow diagrams, as shown in Fig. 4. The designed setup, illustrated in Fig. 3, allows for the operation of each closed aluminum coil separately, two coils together, or all three in series or parallel flow configurations. Additionally, a finned copper coil is installed downstream of the spiral coils to compare the effects of the finned coil with bare spiral coils. The experiments were conducted in a dedicated laboratory with a 60 m^2^ area, equipped with air conditioning and an exhaust system to control ambient temperature and humidity.

The experimental results are categorized into three groups based on the spiral coil heat exchanger: series coils, parallel coils, and finned coils. Initially, pure water was tested, followed by Fe₃O₄-water nanofluid prepared using a two-step process with various mass fractions ranging from 1.5 % to 15 % in six increments. The ambient temperature and inlet humidity were set to 30 °C and 30 %, respectively, under windless conditions due to laboratory constraints. A critical aspect of the experiment is the use of an adiabatic saturator at the air inlet. Air enters the cooling tower through a fully wetted porous environment, cooling to the adiabatic saturation temperature or thermodynamic wet bulb temperature of the inlet air.

**Fig. 9:**
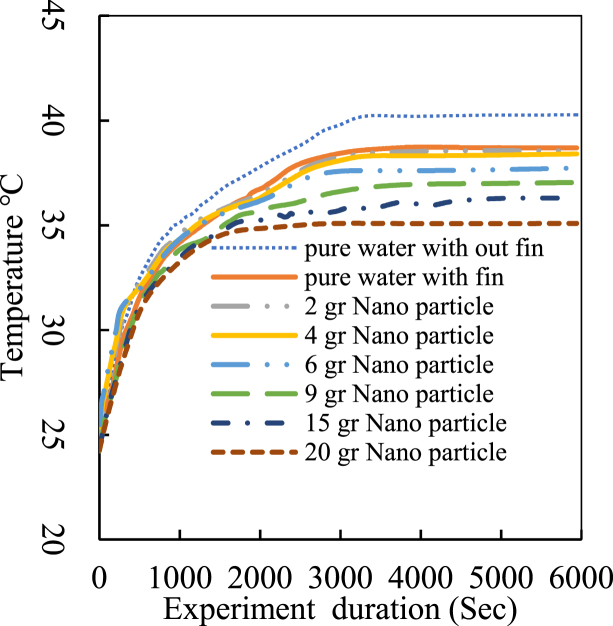
Cooling tower inlet temperature.

Each experiment lasted approximately 1 h and 40 min, reaching a steady state after 30 min. Data were logged at second intervals using an Arduino temperature data logger specifically designed and programmed for this research, resulting in about 5000 data points per graph. Consequently, the graphs appear as continuous lines rather than discrete points, as seen in other experimental results. Fig. 9 depicts the working fluid temperature at the inlet throughout the experiment, comparing pure water with Fe₃O₄-water nanofluid at different mass fractions from 1.5 % to 15 %. The digital thermostat was set to 40 °C with a cutoff temperature of 2 °C. The initial working fluid temperature throughout the cycle was set to 24 °C, matching the ambient temperature.

**Fig. 10:**
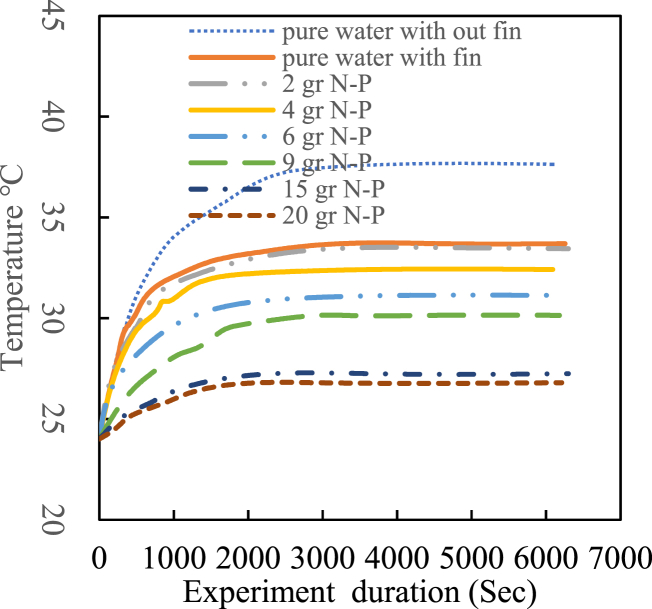
Cooling tower outlet temperature.

**Fig. 11:**
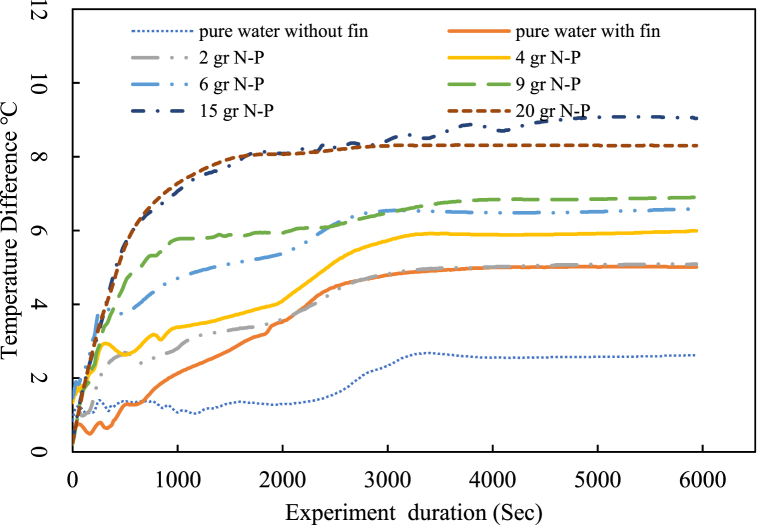
Cooling tower range.

Upon activating the electric heater, there was a significant temperature increase at both the cooling tower inlet and outlet. Results show that the maximum inlet temperature occurred with pure water and decreased with increasing nanofluid mass fraction. The most stable temperature profile was associated with the nanofluid containing a 15 % mass fraction of Fe₃O₄ nanoparticles, while other mass fractions exhibited temperature fluctuations. Furthermore, the steady state was achieved after 30 min for the 15 % nanofluid, compared to about an hour for pure water. Temperature stability in finned coils was significantly higher than in non-finned paths. The results indicate that the highest temperature for nanofluid with 20 g of nanoparticles was 15 % lower than that of pure water. The fin effect reduced the highest temperature by approximately 5 %, even though the finned tube length was eight times shorter than that of the non-finned tubes. Fig. 10, Fig. 11 illustrate the cooling tower outlet temperature and the cooling tower range, respectively, defined as the difference between inlet and outlet temperatures.

**Fig. 12:**
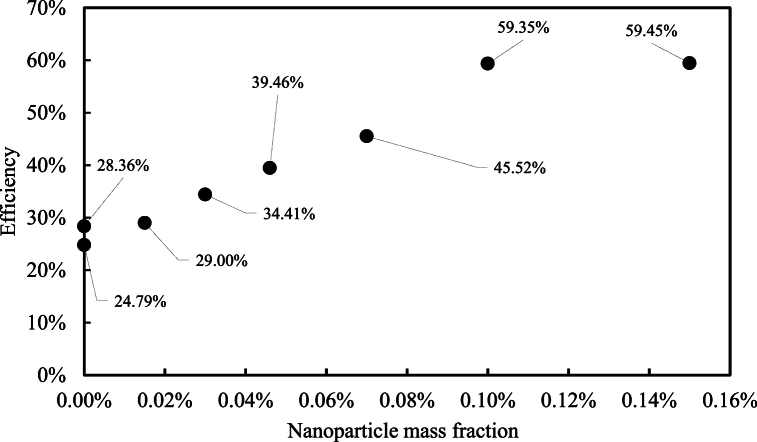
Cooling tower efficiency.

Fig. 12 compares the cooling tower efficiency for pure water and nanofluid with various mass fractions.

In further experiments designed to compare the effect of flow path on cooling tower efficiency, the mass fraction and flow rate were set at 15 % and 6 L/min, respectively. The results, summarized in Table 6, show that in cases 1 to 4, the maximum inlet temperature reached 42 °C, triggering the thermostat to stop the experiment due to insufficient cooling tower capacity. In contrast, stable conditions were achieved in cases 5 to 8, where the inlet temperature did not reach the thermostat limit. The highest efficiency was observed in case 10, where all three coils and the finned tube were in series, followed by the parallel path configuration.

**Table 6:** Cooling tower efficiency at different flow paths.

Case study	Flow path	Equivalent length(m)	T_max_ inlet(°C)	T_max_ outlet(°C)	Cooling tower range(°C)	Cooling tower efficiency (%)
1	Spiral coil 1 & finned tube	18	42	37.3	4.7	22
2	Spiral coil 2 & finned tube	12	42	37.3	4.7	22
3	Spiral coil 3 & finned tube	8	42	37.3	4.7	22
4	Spiral coil 1 & 2 in series flow	30	42	37.3	4.7	22
5	Spiral coil 2 & 3 in series flow	20	37.5	32	5.5	33
6	Spiral coil 1 & 3 in series flow	26	41	36.7	4.3	22
7	Spiral coil 1 & 2 in parallel flow	15	42	–	–	–
8	Spiral coil 2 & 3 in parallel flow	10	42	–	–	–
9	Spiral coil 1 & 3 in parallel flow	13	42	–	–	–
10	Spiral coil 1 & 2 & 3 in series flow with finned coil	41	35.1	26.82	8.28	59
11	Spiral coil 1 & 2 & 3 in series flow without finned coil	38	35	27	8	57
12	Spiral coil 1 & 2 & 3 in parallel flow with finned coil	16	38.2	31.53	6.67	39

--: experiments were not stable, and data were not acceptable.

**Fig. 13:**
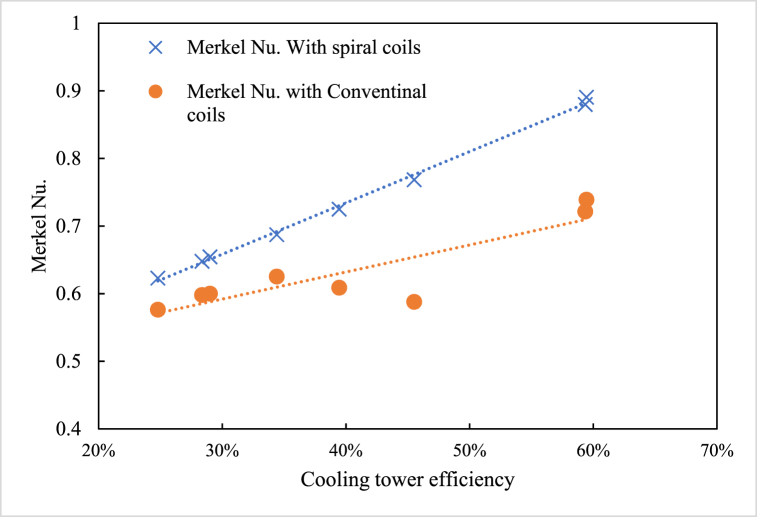
Merkel number vs. cooling tower efficiency.

Merkel number, a dimensionless parameter characterizing heat and mass transfer performance in cooling towers, is shown to have a linear relationship with cooling tower efficiency (Fig. 13). The spiral coils in the hybrid cooling tower promote secondary flows, which can disrupt the boundary layer. This transition from laminar to turbulent flow enhances mixing within the nanofluid, resulting in higher convective heat transfer coefficients. The higher slope of the Merkel number for spiral coils suggests that as efficiency increases, the impact of using spiral coils becomes even more significant compared to conventional coils. The spiral design provides better heat exchange due to increased turbulence and improved surface contact, making it more suitable for applications where high-performance cooling is required.

**Fig. 14:**
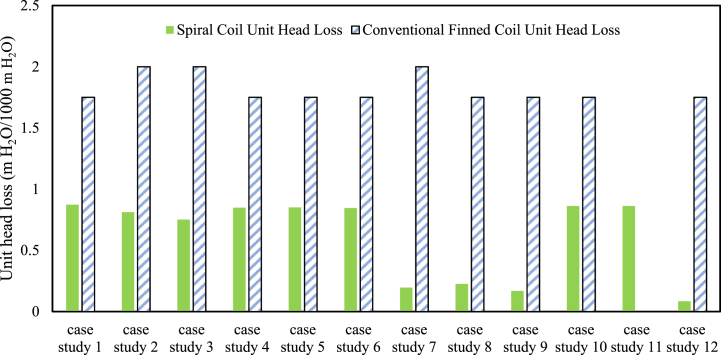
Pressure drop comparison between conventional finned coil and spiral coils.

Fig. 14 shows that the pressure drop in the conventional finned coil was almost twice that of the spiral coils. However, case 10 exhibited the highest pressure drop due to the longest pipe length, while the parallel path demonstrated the best hydraulic performance. Pressure-drop and pump power were analyzed using pressure gauges at the cooling tower inlet and outlet, along with the hydraulic modeling with hydraulic analysis software, EPANET.

**Fig. 15:**
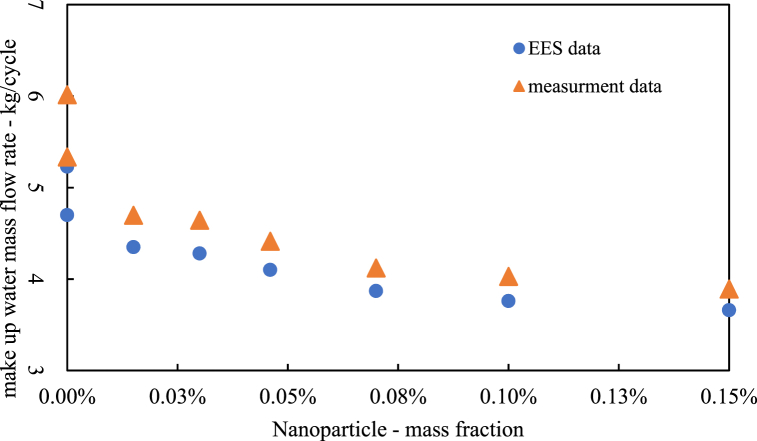
Cooling tower evaporation rate.

Another significant result is the ability to measure the evaporation rate using a flow meter at the makeup water inlet and calculate the vaporization amount based on the humidity difference between the air inlet and outlet. The setup was designed to minimize droplet waste due to fan effects, utilizing a PVC pipe with 250 holes aligned to the fan axis to reduce water loss. Fig. 15 compares measured and simulated data for evaporation rates, showing that increased nanoparticle mass reduces evaporation rates in the open section, aligning with simulation data. The quality of water in the closed part of the system remains constant, with neither scaling nor evaporation, and the nanoparticle mass fraction in the working fluid stays stable.

## 6 Conclusion

This experimental research utilized Fe₃O₄-water nanofluid in various concentrations to investigate the efficiency of a hybrid cooling tower comprising three coaxial spiral coils and a conventional copper finned coil. A key aspect of this design is the isolation of the closed system from the evaporation part of the cooling tower, ensuring that nanoparticles do not settle in the basin or escape due to fan effects. By modifying the flow diagram, both the evaporation rate and nanoparticle loss were significantly reduced.

The findings reveal that the nanofluid reaches full homogenization 90 min after the tests commence. The conduction heat transfer coefficient of the nanofluid during the cycle is 10 % higher than in the ultrasonicated state and 58 % higher than in the settled state. From an analytical perspective, the Merkel number plays a crucial role in the efficiency of a hybrid cooling tower. This study shows that in a hybrid cooling tower, which combines wet and dry cooling mechanisms, the Merkel number is influenced by factors such as water flow rate, air flow rate, tower geometry, fill material and operating conditions. The spiral coil geometry notably increased the Merkel number by an average of 8 %.

The most stable temperature profile was achieved with a 15 % mass fraction of Fe₃O₄ nanoparticles. The use of spiral coils induced secondary flow, which enhanced the convection heat transfer coefficient. Increasing the nanoparticle concentration in the main fluid improved the cooling tower's efficiency by up to 59 % compared to pure water. The maximum temperature reduction for the nanofluid with 20 g of nanoparticles was 15 % lower than that of pure water, with the lowest cooling tower outlet temperature recorded at 26.5 °C for the 15 % nanoparticle mass fraction.

The combination of turbulent flow and enhanced thermal properties of Fe₃O₄-water nanofluid resulted in substantial increases in cooling tower efficiency.

Reducing the evaporation rate in conventional cooling towers is crucial for two main reasons:

1. **Water Conservation:** Reducing water consumption by 27 % compared to conventional wet cooling towers.
2. **Enhanced Heat Transfer:** Increasing overall heat transfer efficiency in hybrid cooling towers with secondary flow by up to 59.45 %.

This study underscores the benefits of using Fe₃O₄-water nanofluid and optimized flow configurations by means of spiral coils, to enhance the performance and sustainability of hybrid cooling towers.

## 7 Future exploration

There are several potential challenges and limitations that need to be addressed in future research.

**Flow distribution in spiral coils**: The secondary flow in the spiral coils enhances heat transfer, but ensuring uniform flow distribution across **large-scale systems** may be difficult. As the system scales up, maintaining consistent flow and preventing areas of low circulation or stagnation could require more sophisticated flow management designs, such as optimized pipe geometries or additional flow control mechanisms.

**Scale-up challenges**: The transition from a laboratory-scale system to industrial applications introduces challenges. Structural integrity needs to be carefully studied in larger installations.

**Velocity variation**: In spiral coils, flow velocity plays a crucial role in determining the heat transfer rate. If the flow velocity is too low, the benefits of the secondary flow could be minimized, whereas high flow rates could increase wear and tear on the system and lead to mechanical failures. Fine-tuning flow velocity is necessary to maintain efficient heat transfer without increasing operational risks.

**hybrid nanofluids:** The use of hybrid nanofluids and the effects of magnetic fields on Fe₃O₄ nanoparticles present promising avenues for future studies in hybrid cooling towers. Magnetic nanoparticles like Fe₃O₄ have unique properties that can be influenced by external magnetic fields, potentially further enhancing heat transfer performance.

## CRediT authorship contribution statement

**Danial Fallah Heravi:** Writing – review & editing, Writing – original draft, Methodology, Formal analysis, Data curation, Conceptualization. **Hamid Reza Goshayeshi:** Writing – review & editing, Validation, Supervision, Project administration, Methodology, Formal analysis, Data curation, Conceptualization. **Reza Saleh:** Writing – review & editing, Validation, Project administration, Methodology, Formal analysis, Data curation, Conceptualization.

## Data and code availability

The data that has been used is confidential.

The author team have chosen not to share the data at this time as it will be utilized for future research publications. We appreciate your understanding.

## Declaration of competing interest

The authors declare that they have no known competing financial interests or personal relationships that could have appeared to influence the work reported in this paper.
